# Intra-hepatic splenosis as an unexpected cause of a focal liver lesion in a patient with hepatitis C and liver cirrhosis: a case report

**DOI:** 10.4076/1757-1626-2-8335

**Published:** 2009-08-19

**Authors:** Marianne Menth, Karin Herrmann, Alexander Haug, Bijan Raziorrouh, Reinhart Zachoval, Christina-Maria Jung, Carsten Otto

**Affiliations:** 1 Medical Department 2, University of Munich - GrosshadernMarchioninistrasse 15, D-81377 MunichGermany; 2 Institute of Clinical Radiology, University of Munich - GrosshadernMarchioninistrasse 15, D-81377 MunichGermany; 3 Department of Nuclear Medicine, University of Munich - GrosshadernMarchioninistrasse 15, D-81377 MunichGermany

## Abstract

**Introduction:**

Splenosis is the heterotopic autotransplantation of splenic tissue, mostly found after splenic trauma or surgery in the abdominal, pelvic or thoracic cavity. Here we report a patient with a history of splenectomy after polytrauma with chronic hepatitis C and liver cirrhosis presenting with an hepatic mass of unknown origin.

**Case presentation:**

The lesion could not be exactly classified by ultrasound, computed tomography, angiography and biopsy, classical features of malignancy were not fulfilled, and on the other hand a neoplastic process could neither be excluded. After revision of a MRI performed in our centre it appeared that the liver mass contrasted in the same way as the remaining accessory spleens in the left upper quadrant. A selective Tc-99m-labelled heat-denatured autologous red blood cells scintigraphy of the spleen was performed and showed both the accessory spleens in the left upper quadrant and spleen-typical tissue in projection to the left liver lobe and confirmed the diagnosis of splenosis.

**Conclusion:**

Although intrahepatic splenosis represents an extremely rare condition, this diagnosis should always be taken into consideration in patients with history of abdominal trauma with splenic involvement presenting with an indeterminate focal liver lesion. The diagnosis of splenosis may then be reliably confirmed by Tc-99m-DRBC scintigraphy.

## Introduction

Intrahepatic masses occurring in patients with liver cirrhosis and hepatic viral infections have a high likelihood to be regenerative nodules or HCC. The differential diagnosis of benign pseudo-lesions may not be obvious in this context and comparably rare. Diagnostic imaging such as ultrasound, liver MRI and transfemoral arterial angiography play a pivotal role but may not always be conclusive at first glance. Diagnostic interpretation in the clinical context is always warranted.

We report a case of intra-hepatic splenosis in a patient with hepatitis C, liver cirrhosis and a history of severe abdominal trauma with multi-organ contusion and splenic rupture followed by splenectomy. The value of various diagnostic modalities in this rare distinct pathology is discussed.

## Case presentation

A 43-year-old Caucasian man from Germany with liver cirrhosis and chronic hepatitis C virus infection was referred to our tertiary centre presenting with a tumour in the left liver lobe of indeterminate origin. Infection with HCV occurred in 1979, following multiple transfusions in the context of a severe polytrauma with liver rupture, diaphragmatic rupture, left kidney rupture and splenic rupture, the latter requiring subsequent splenectomy. In 2001, a liver biopsy was performed confirming the presence of liver cirrhosis. The hepatitis C genotype was 1a according to Simmonds. Antiviral therapy was started immediately with pegylated interferon and ribavirin but was not successful due to virus relapse at the end of the therapy.

In October 2004, a second try of antiviral therapy was planned. Routine ultrasound of the liver at that time revealed a polylobulated mass with regular margins in segment II of the left liver lobe. Subsequent abdominal MRI confirmed the presence of multiple confluent nodular lesions in the left liver lobe measuring 2.5 × 7.0 cm. These lesions showed hypervascularity on contrast-enhanced liver MRI and malignancy was suspected. The patient was admitted to our hospital to confirm the diagnosis and to establish appropriate treatment of suspected HCC.

At admission, the patient did not complain about any symptoms except for increased fatigue. He did not suffer from weight loss, fever or nocturnal sweats. The physical examination was normal. Clinically, the cirrhosis was graded Child A. Serum aminotransferases were slightly elevated (GPT 95 U/l, GOT 80 U/l). AFP was normal with 6.4 ng/ml.

**Figure 1.**
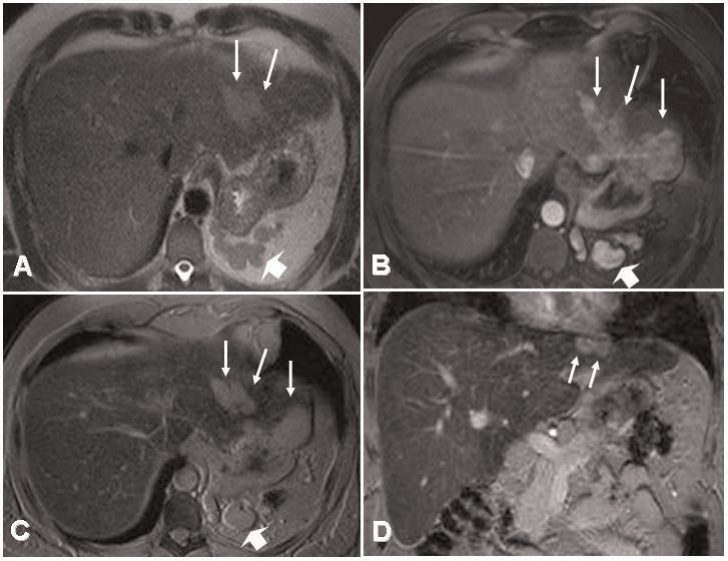
MRI of the liver. T2-weighted single-shot turbo spinecho imaging (HASTE) MRI of the liver shows slightly hyperintense polylobular lesion in the left lobe of the liver, segment II **(A, arrows)**. Multiple nodular structures are identified in the splenic recess indicating recurrent splenic tissue after splenectomy (large arrow). After intravenous application of Gadolinium-DTPA (Magnevist, Schering, Berlin, Germany), these lesions exhibit marked contrast enhancement in the early arterial phase, as it may also be seen in the case of hepato-cellular carcinoma **(B)**. With hepato-specific contrast agents (iron-oxide particles, Resovist^®^, Schering, Berlin, Germany), the hepatic lesion lacks iron uptake, which is shown on axial and coronal T2*-weighted images **(C, D)** and is indicative of the presence of non-hepatic tissue. Especially the coronal images delineate the indenting nature of the space-occupying lesion into the hepatic tissue and the close relationship to the diaphragm, suggesting an extrahepatic location of the lesion **(D)**.

MRI was repeated in double contrast technique including dynamic imaging after intravenous application of standard Gadolinium-DTPA (0.1 mmol/kg, Magnevist®, Bayer-Schering Healthcare, Berlin, Germany) and SPIO - enhanced imaging in the same examination (Resovist®; Bayer-Schering Healthcare, Berlin, Germany). Besides the signs of subtle micro-nodular regenerative liver cirrhosis of the entire liver parenchyma, multiple nodular lesions between 4 and 36 mm in diameter were identified in the subcapsular region of segment II. The partially confluent nodules showed pronounced slightly inhomogeneous hypervascularity in the early arterial phase of dynamic Gd-enhanced liver MRI and a lack of contrast uptake of the liver specific superparamagnetic iron-oxide (SPIO) containing contrast agent (Figure 1).

Enlarged paracaval and hilar lymph nodes and accessory splenic tissue in the left subdiaphragmatic area were identified at the same examination. The suspicion of a potentially malignant process concerning the hepatic nodules was sustained and further investigation was considered necessary to confirm or rule out hepatocellular carcinoma (HCC). Transfemoral intraarterial angiography of the liver was performed as a primarily diagnostic and potentially therapeutic procedure. It showed regular branches of the hepatic artery but no pathologic vessels or parenchymal foci of hypervascularity.

CT-guided biopsy was then considered and attempted although the peripheral location of the nodules offered limited approach. The histologic findings showed cirrhotic liver tissue with chronic periportal inflammation and steatosis of up to 30 % of the hepatocytes. There was no confirmation of malignancy.

Despite the negative results of all diagnostic and imaging modalities, the potential malignancy of the lesions could still not be ruled out at this point. Therefore, the MRI which was performed in our center, and all other investigations were revised for a second time.

**Figure 2.**
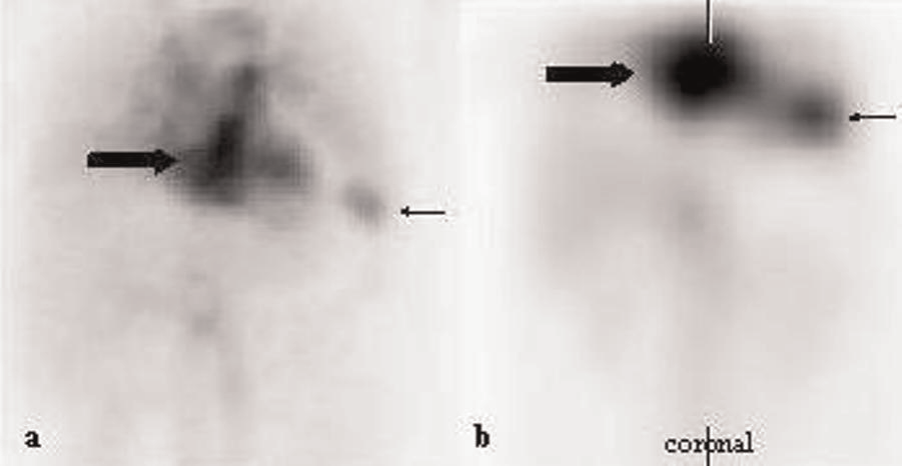
Tc-99m-DRBC scintigram. Heat-damaged and Tc-99 m-labeled autologous red blood cell scintigram shows uptake of the labeled cells in the left lobe of the liver (thick arrow) and in the vicinity of the removed spleen (thin arrow) in the planar scan **(a)** and the SPECT-images **(b)** corresponding to the masses seen in MRI and CT.

Facing a similar arterial contrast enhancement behaviour of the intrahepatic nodules on MRI when compared to the residual and accessory splenic tissue observed in the left subdiaphragmatic region, a selective scintigraphy with Tc-99m-labelled heat-denatured autologous red blood cells (Tc-99m-DRBC) was performed in the attempt to investigate also for the rare differential diagnosis of intra-hepatic splenosis in this context. Tc-99m-DRBC finally confirmed this differential diagnosis showing uptake in both of the accessory spleens in the left upper quadrant and in the area projecting to the left liver lobe (Figure 2). A follow-up MRI after nine months revealed no change and identical findings with respect to all nodular lesions and the diagnosis of intrahepatic splenosis was established. As there was no need for further treatment with regards to the splenosis, the patient has restarted therapy with pegylated interferon and ribavirine.

## Discussion

Splenosis is the heterotopic autotransplantation of splenic tissue and occurs in 16 to 67% of the patients after traumatic spleen rupture or spleen surgery [1]. It is important to distinguish splenosis from accessory spleens. Accessory spleens are located on the left side of the dorsal mesogastrium in the region of the splenopancreatic or gastrosplenic ligaments. They are supplied by a branch of the splenic artery. In contrast, splenic implants are supplied by small arteries of the surrounding tissue penetrating the surrounding capsule [1,2].

Splenosis is a rare entity. To date, around 100 cases were reported in the literature until 2004 [3]. However, only very few cases with isolated hepatic localisation were described [3,4]. The most frequent sites of implanted splenic tissue spread after trauma are the abdominal or pelvic cavity. Intrathoracic sequestration has been observed in patients with abdominal trauma penetrating the diaphragm. Although the diaphragm was ruptured also in our patient during the trauma, no other site of implantation was identified.

Hematogenous spread of splenic pulp is supposed to be a possible mechanism of seeding into the liver. The subcapsular location of the splenic tissue in our case may suggest the implantation on the inferior surface of the diaphragm which was ruptured in the same trauma mechanism rather than by hematogenous spread into the liver. However, the marked indentation of the nodules into the liver parenchyma made exact localization difficult.

In the vast majority of cases with splenosis, patients are asymptomatic and an undetermined mass is found incidentally. Sometimes, non-specific symptoms such as abdominal pain or diarrhea may lead to the diagnosis. Gastrointestinal bleeding after rupture of a splenic implant nodule and intestinal obstruction are rather rare complications associated with splenosis.

Chronic hepatitis C has a prevalence of about 1% in the Middle-European population. Transmission of the hepatitis used to occur frequently after transfusion in polytraumatized patients. 20% of the patients develop liver cirrhosis. The incidence of HCC in patients with liver cirrhosis and hepatitis C is about 7 % in 5 years [5]. Thus, any hepatic mass in a patient with hepatitis C and liver cirrhosis should always raise suspicion of HCC.

The spectrum of differential diagnosis for focal liver lesions in diagnostic radiologic imaging is wide but may be narrowed down in patients with hepatitis C and liver cirrhosis to hemangioma, adenoma, focal nodular hyperplasia, regenerative nodules, dysplastic nodules and hepatocellular carcinoma.

The EASL (European Association of the Study of the Liver) criteria for the diagnosis of HCC recommend a coincidental presence of arterial hypervascularity of a suspect nodule in two imaging techniques or an AFP level over 400 ng/ml and concomitant arterial hypervascularity in one imaging technique. According to Bolondi, in a study of small nodules in patients with liver cirrhosis, 38 % of the HCC nodules measuring 1-2 cm and 16 % of the HCC nodules measuring 2-3 cm were not satisfying the EASL criteria concerning hypervascularity. All nodules larger than 2 cm resulted to be HCC [6]. Hence, the absence of hypervascularity is not necessarily contradictory to the suspicion of malignancy.

The respective nodules in our patient showed slightly inhomogeneous but marked contrast enhancement in the early arterial phase of dynamic imaging after Gadolinium-DTPA, evocative of HCC. The additional lack of uptake of the SPIO contrast agent further supported the suspicion of malignancy.

Double contrast MRI of the liver using Gadolinium-DTPA and SPIO as liver specific contrast agent has proven to enhance the diagnostic accuracy in the detection and characterization of focal liver lesions and HCC [7] and is widely accepted as state-of-the art imaging. Due to the presence of reticuloendothelial system, splenic tissue is supposed to present some uptake of the iron particles. Hence, signal void should be observed in splenic tissue to a certain degree. In our case, iron uptake was completely absent both in the accessory spleen and in the intrahepatic nodules of splenosis, leading to confounding results and misleading image interpretation.

The liver biopsy performed in our centre showed cirrhotic tissue and did not reveal any components of splenic tissue. As a consequence, it has to be assumed that the lesion of interest was missed at biopsy. The limited access to the lesion in its subcapsular and subdiaphragmatic location in the left upper abdominal quadrant may be the most plausible explanation to this negative result. According to the literature, in CT-guided liver biopsies the diagnostic yield is 67 to 96 % [8,9].

It remains an interesting observation that a large number of the reported cases of hepatic splenosis are patients with hepatitis B or C with or without liver cirrhosis [3]. It could be speculated that these patients have a specific vulnerability concerning splenosis. However, it seems more likely that these patients are better investigated with imaging methods than otherwise healthy people.

The specific diagnosis of splenosis is most reliably established with help of Tc-99m-DRBC scintigraphy. Tc-99m-DRBC scintigraphy has shown to be more sensitive than Tc-99m-sulfur colloid scintigraphy [10]. About 90% of the administered Tc-99m-labeled heat-damaged autologous red blood cells are sequestered by reticuloendothelial cells and can be used to identify splenic tissue.

As splenosis is a quite rare disease there exist no studies with a larger cohort of patients to evaluate sensitivity and specificity of Tc-99m-DRBC scintigraphy. Yet, Tc-99m-DRBC scintigraphy is still considered as the gold standard in the non-invasive diagnosis of splenosis.

## Conclusion

Although intrahepatic splenosis represents an extremely rare condition, this diagnosis should always be taken into consideration in patients with history of abdominal trauma with splenic involvement presenting with an indeterminate focal liver lesion.

At contrast enhanced MRI with Gadolinium-chelates, splenosis shows a signal behaviour and enhancement pattern similar to that of potentially present accessory spleen but does not necessarily exhibit iron uptake on SPIO enhanced imaging. The diagnosis of splenosis may then be reliably confirmed by Tc-99m-DRBC scintigraphy and will help to avoid unnecessary major surgery or other invasive therapeutic procedures.

Splenosis per se does not require particular follow up with diagnostic imaging or other investigations. Interferon therapy of hepatitis C in the cirrhotic patient with splenosis is safe, however, careful follow-up is crucial.

## Abbreviations

AFP: alpha-fetoprotein
CT: computed tomography
DRBC: heat-denatured autologous red blood cells
DTPA: diethylenetriamine penta-acetic acid
EASL: European Association of the Study of the Liver
GOT: glutamate-oxalacetate-transaminase
GPT: glutamate-pyruvate-transaminase
HCC: hepatocellular carcinoma
HCV: hepatitis C virus
MRI: magnetic resonance imaging
SPIO: super-paramagnetic iron oxide
Tc: Technetium
